# Pivotal trial of a deep-learning-based retinal biomarker (Reti-CVD) in the prediction of cardiovascular disease: data from CMERC-HI

**DOI:** 10.1093/jamia/ocad199

**Published:** 2023-10-17

**Authors:** Chan Joo Lee, Tyler Hyungtaek Rim, Hyun Goo Kang, Joseph Keunhong Yi, Geunyoung Lee, Marco Yu, Soo-Hyun Park, Jin-Taek Hwang, Yih-Chung Tham, Tien Yin Wong, Ching-Yu Cheng, Dong Wook Kim, Sung Soo Kim, Sungha Park

**Affiliations:** Division of Cardiology, Severance Cardiovascular Hospital, Yonsei University College of Medicine, Seoul 03722, South Korea; Ocular Epidemiology Research Group, Singapore Eye Research Institute, Singapore National Eye Centre, Singapore 169856, Singapore; Mediwhale Inc, Seoul 08378, South Korea; Division of Retina, Severance Eye Hospital, Yonsei University College of Medicine, Seoul 03722, South Korea; Department of Ophthalmology and Visual Science, University of Texas Health Science Center at Houston, Houston, TX 77030, USA; Mediwhale Inc, Seoul 08378, South Korea; Ocular Epidemiology Research Group, Singapore Eye Research Institute, Singapore National Eye Centre, Singapore 169856, Singapore; Food Functionality Research Division, Korea Food Research Institute, Wanju 55365, South Korea; Food Functionality Research Division, Korea Food Research Institute, Wanju 55365, South Korea; Department of Food Biotechnology, University of Science and Technology, Daejeon 34113, South Korea; Ocular Epidemiology Research Group, Singapore Eye Research Institute, Singapore National Eye Centre, Singapore 169856, Singapore; Centre for Innovation and Precision Eye Health, and Department of Ophthalmology, Yong Loo Lin School of Medicine, National University of Singapore, Singapore 117597, Singapore; Ocular Epidemiology Research Group, Singapore Eye Research Institute, Singapore National Eye Centre, Singapore 169856, Singapore; Tsinghua Medicine, Tsinghua University, Beijing 100084, China; Ocular Epidemiology Research Group, Singapore Eye Research Institute, Singapore National Eye Centre, Singapore 169856, Singapore; Centre for Innovation and Precision Eye Health, and Department of Ophthalmology, Yong Loo Lin School of Medicine, National University of Singapore, Singapore 117597, Singapore; Department of Information and Statistics, Department of Bio & Medical Big Data, Research Institution of National Science (RINS), Gyeongsang National University, Jinju 52828, South Korea; Division of Retina, Severance Eye Hospital, Yonsei University College of Medicine, Seoul 03722, South Korea; Division of Cardiology, Severance Cardiovascular Hospital, Yonsei University College of Medicine, Seoul 03722, South Korea

**Keywords:** regulated pivotal study, deep learning, software as a medical device (SaMD), cardiovascular disease, retinal photograph, Reti-CVD

## Abstract

**Objective:**

The potential of using retinal images as a biomarker of cardiovascular disease (CVD) risk has gained significant attention, but regulatory approval of such artificial intelligence (AI) algorithms is lacking. In this regulated pivotal trial, we validated the efficacy of Reti-CVD, an AI-Software as a Medical Device (AI-SaMD), that utilizes retinal images to stratify CVD risk.

**Materials and Methods:**

In this retrospective study, we used data from the Cardiovascular and Metabolic Diseases Etiology Research Center-High Risk (CMERC-HI) Cohort. Cox proportional hazard model was used to estimate hazard ratio (HR) trend across the 3-tier CVD risk groups (low-, moderate-, and high-risk) according to Reti-CVD in prediction of CVD events. The cardiac computed tomography-measured coronary artery calcium (CAC), carotid intima-media thickness (CIMT), and brachial-ankle pulse wave velocity (baPWV) were compared to Reti-CVD.

**Results:**

A total of 1106 participants were included, with 33 (3.0%) participants experiencing CVD events over 5 years; the Reti-CVD-defined risk groups (low, moderate, and high) were significantly associated with increased CVD risk (HR trend, 2.02; 95% CI, 1.26-3.24). When all variables of Reti-CVD, CAC, CIMT, baPWV, and other traditional risk factors were incorporated into one Cox model, the Reti-CVD risk groups were only significantly associated with increased CVD risk (HR = 2.40 [0.82-7.03] in moderate risk and HR = 3.56 [1.34-9.51] in high risk using low-risk as a reference).

**Discussion:**

This regulated pivotal study validated an AI-SaMD, retinal image-based, personalized CVD risk scoring system (Reti-CVD).

**Conclusion:**

These results led the Korean regulatory body to authorize Reti-CVD.

## Graphical Abstract

**Figure:**
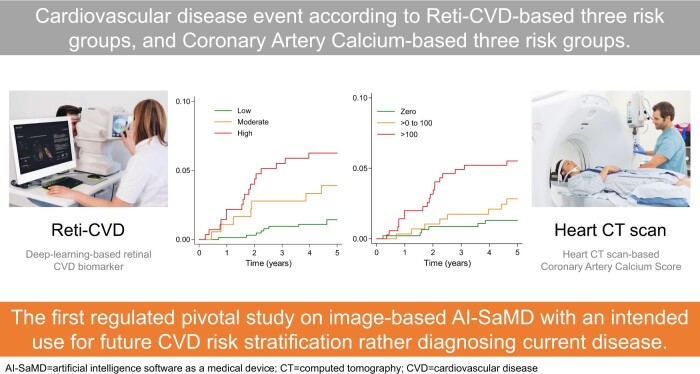

## Introduction

Cardiovascular disease (CVD) is the leading cause of death worldwide.^1^ Traditional risk factors, such as presence of hypertension, diabetes mellitus, smoking, and hypercholesterolemia, have led to the development of CVD risk prediction models and to guide developments in prevention and therapy.^2^ However, up to 20% of patients with coronary disease have none of these traditional risk factors, and 40% have only one.^3^ The broad implementation and use of such CVD risk prediction models based on traditional risk factors alone are therefore restricted.^4^^,^^5^

From a public health and clinical perspective, the primary goal of CVD risk stratification is dependent on accurately identifying and triaging high-risk individuals before the development of symptomatic CVD events. As a result, an increasing number of novel biomarkers have been evaluated to predict CVD events; this includes cardiac computed tomography (CT)-measured coronary artery calcium (CAC) score, carotid intima-media thickness (CIMT), and brachial-ankle pulse wave velocity (baPWV). Among these new biomarkers, CAC is a promising preclinical marker of atherosclerosis and is strongly associated with CVD risk.^6^ Therefore, in the 2018 American College of Cardiology/American Heart Association (ACC/AHA) guidelines, CAC score has been recommended as an additional test to refine risk estimates for statin treatment initiation.^7^

Retinal photographic imaging is an increasingly available, relatively simple, effective, and non-invasive tool that provides fast and accurate information about the human vasculature in the retina. The presence of overt retinal vascular damage (eg, microaneurysms, retinal hemorrhages) is clinically recognized as an indicator of target organ damage associated with hypertension.^8^^,^^9^ Even more subtle changes in the retina that may not even be visible to the human eye can potentially be detected with the advent of artificial intelligence (AI) and deep learning.^10–12^

Previously, we developed an AI-deep-learning algorithm to predict the probability of presence of CAC, and created a 3-tier CVD risk stratification system that allowed us to stratify CVD risk, which we tested in the UK Biobank and a diversified Asian population.^13–15^ We have further refined this algorithm (referred to as Reti-CVD) to be commercialized as an AI-Software as a Medical Device (AI-SaMD). Regulatory agencies in different countries have now started to approve a range of AI-SaMD products; for example, South Korea has successfully established a regulatory framework for AI-SaMD,^16^ and the Korean Ministry of Food and Drug Safety (K-MFDS) has authorized approximately 150 AI-SaMDs for a variety of clinical use.

Reti-CVD is different from typical AI-SaMDs, which are currently actively being approved and purpose for diagnosing current diseases such as diabetic retinopathy. Reti-CVD is an AI-SaMD that quantifies future CVD risk and provides risk as 3-tier groups. Therefore, the study design and regulatory process for obtaining approval were unique. To advance the concept that retina imaging is a possible tool for CVD risk stratification, we conducted this regulated pivotal trial in which we evaluated the efficacy of Reti-CVD as an AI-SaMD, using a single-center conformity-design, confirmatory retrospective analysis.

## Methods

### Ethics statement

Our study was approved by the Severance Hospital Institutional Review Board (IRB). This study adhered to the tenets of the Declaration of Helsinki, complied with the applicable policies regarding the ethical conduct of human subject research and medical devices (Korean Good Clinical Practice, KGCP; International standard ISO14155). Written informed consent was obtained from the participants of the original study.

In South Korea, medical devices with marked improvement in safety and effectiveness over existing ones have been designated as “innovative medical devices”,^16^ which is similar to the “breakthrough devices program” in the US Food & Drug Administration (FDA). The Reti-CVD was designated as the innovative medical device and the study design of this “K-MFDS-regulated pivotal trial” was approved from the K-MFDS before commencement of IRB process. The K-MFDS actively engages in clinical trial design verification together with the device company prior to clinical trial, minimizing errors, and speeding up the process.

### Study population

We conducted a retrospective analysis using prospectively collected single-center clinical data of “Cardiovascular and Metabolic Diseases Etiology Research Center-High Risk (CMERC-HI) Cohort” (Severance Hospital IRB Approval No. 4-2013-0581). The CMERC-HI was funded by the Ministry of Health and Welfare of the Korean Government in 2013, and enrolled participants between November 18, 2013, and June 29, 2018. The goal was to identify novel risk factors and biomarkers for CVD. The original study of CMERC-HI Cohort was registered as a “prospective” observational study on May 13, 2014 (NCT02003781).

This previously collected data had all the information (eg, retinal photos, CVD outcomes, etc.) necessary to validate our Reti-CVD, and we retrospectively analyzed the data as secondary research. This pivotal study was registered as a “retrospective” observational study on February 17, 2022 (KCT0007047). We analyzed the data of participants who previously agreed to secondary use of data for research purposes.

The initial Proof of Concept (PoC) study on Reti-CVD used the interim CMERC-HI dataset spanning 2013-2016 as one of the validation sets. Conversely, our current investigation employs a comprehensive dataset extending up to 2020. We assess the efficacy of Reti-CVD in comparison with CAC, CIMT, and baPWV in prediction of CVD events (detailed in Appendix S1).

The retinal images of enrolled patients were evaluated by Reti-CVD and its performance was validated in prediction of CVD events through comparison with other biomarkers from the CMERC-HI cohort. Retinal images were obtained by a non-mydriatic retinal camera (Visucam NM/FA; Carl Zeiss Meditec, Jena, Germany).

We included patients: (1) enrolled between November 2013 and June 2018; (2) followed-up up to 5 years after enrollment or with follow-up data up until December 31, 2020, or with non-fatal and fatal CVD events within 5 years’ enrollment; and (3) between 20 and 80 years old.

**Figure 1.**
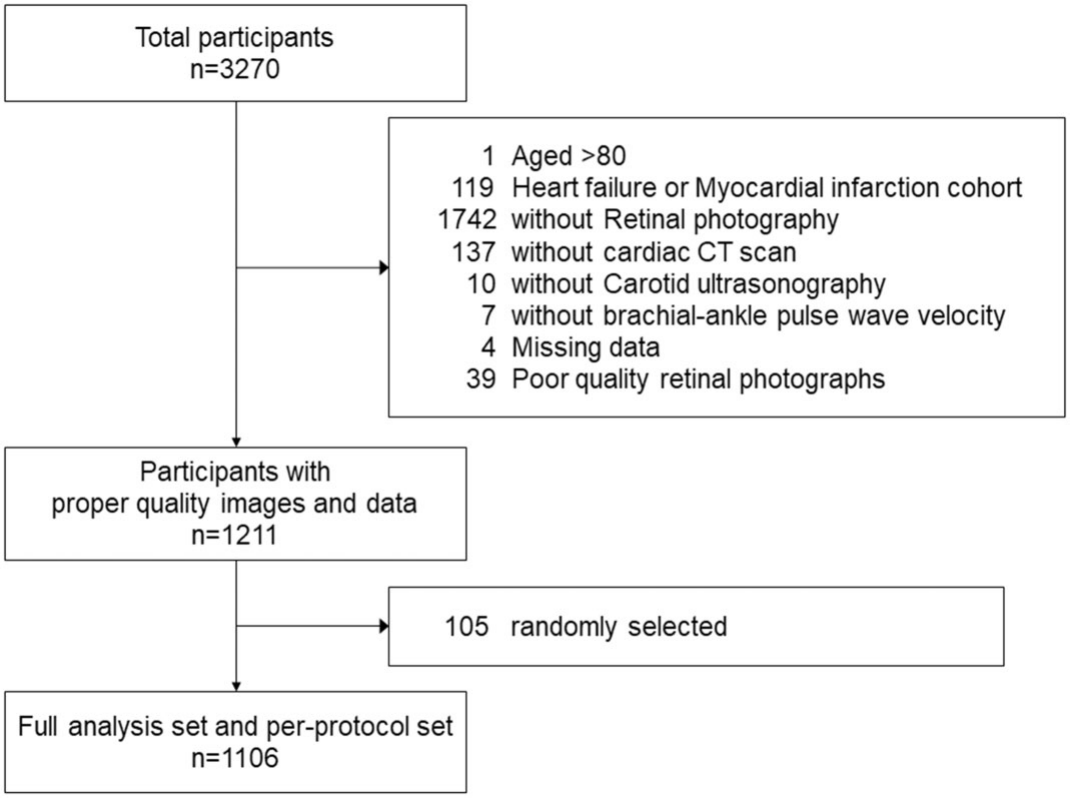
Study flowchart.

We then excluded patients: (1) enrolled in the heart failure/myocardial infarction arms of the CMERC-HI cohort; (2) without retinal fundus imaging; (3) without cardiac CT for calcium scoring; (4) without carotid ultrasonography; (5) without baPWV examination; (6) with missing baseline demographic data including age, sex, smoking history, medical history for hypertension/diabetes/dyslipidemia; (7) with poor quality retinal fundus imaging (ie, media opacity, artifacts) preventing proper assessment by Reti-CVD (Figure 1).

### Definition of CVD events

We defined CVD events as any CVD incidences including heart failure hospitalization, stroke, myocardial infarction, or CVD mortality. In the CMERC-HI cohort, participants were contacted by telephone annually and a review of hospital records to ascertain CVD events, including heart failure, myocardial infarction, and stroke. Detailed definition of CVD events are provided in Appendix S2.

### New retinal-based CVD risk stratification system

Details of deep-learning algorithm regarding model development have been described elsewhere^13^ and a summary is provided in Appendix S3. Briefly, in an earlier study, we developed a deep-learning algorithm using retinal photographs for predicting the presence versus absence of CAC using Korean health screening center data. The probability scores ranged from 0 to 1 (as a continuous variable), with a higher value indicating increased probability of CAC presence, which we referred to as the Reti-CVD score.

This “Reti-CVD score” was designed to calibrate the amount of association between retinal microvascular signs and the presence of CAC, which is a robust predictor of CVD risk. In this earlier study, we found that the Reti-CVD score could predict future CVD events in a longitudinal study rather than just predict CAC in a cross-sectional study. Finally, we confirmed that the Reti-CVD score could stratify future CVD risk in 3 external longitudinal studies (time-to-events data) including Korean, Singaporean Malay/Indian/Chinese, and British White cohorts.^13^

In this regulated pivotal study, Reti-CVD is the product name of an AI-SaMD, and is a retinal image-based, personalized CVD risk scoring tool (ie, Reti-CVD score). To aid healthcare professionals in identifying at-risk patients in primary prevention, Reti-CVD also provides 3-tiers (low-, moderate-, and high-risk) risk groups (ie, Reti-CVD risk groups) based on Reti-CVD score. These 3 risk groups were defined based on pre-specified thresholds (Appendix S3). The probability scores, Reti-CVD score, derived from deep learning indicate the likelihood of CAC presence, ranging from 0 to 1. Based on the distribution of Reti-CVD scores, this system classifies risks into 3 tiers using the tertiles from the CMERC-HI interim data in earlier study.^13^ The aim of risk stratification extends beyond merely allocating a risk score; it seeks to provide a comprehensive and integrated perspective of patients to facilitate appropriate management. Our 3-tiered risk stratification approach is intended to serve as a benchmark, analogous to CAC. This is structured by segmenting it into the 3 risk groups of CAC, as delineated in US guidelines,^7^,^13^ and their respective risk categories.

### Other CVD biomarkers

To compare the performance in prediction of future CVD events, the CIMT, the baPWV, and CAC score obtained through cardiac CT assessment were included for secondary analysis. Detailed measurement methods are described in Appendix S4.

### Statistical analysis

Detailed sample size calculations are provided in Appendix S5. Based on an earlier publication, the sample size of 1050 participants was calculated with a 5% significance level with an assumed hazard ratio (HR) trend of 2.02 and 80% power for non-one HR detection. Among the calculated sample size of 1050 participants, a drop-out rate of 5% was assumed, resulting in the total planned subject number of 1106. Thus, after retrospectively reviewing the cohort data for inclusion/exclusion, a total of 1106 subjects were planned to be enrolled for the analysis. If a greater number of subjects were eligible for enrollment after inclusion/exclusion than the planned sample size, then randomization for further exclusion would be performed to select the target number of 1106 subjects (Appendix S6). In a previous PoC study,^13^ the interim result was presented using only a part of participants in CMERC-HI (*n* = 527). Herein, we included 1106 CMERC-HI participants based on sample size calculation. Randomization for further exclusion was done using SAS Version 9.4. Analyses were done using *P* <.05 as the significance level and using Stata/MP version 14.0 for survival analysis.

### Primary efficacy endpoints

We hypothesized that the retinal fundus photograph-based risk assessment SaMD, “Reti-CVD” will be able to differentiate individuals into a 3-tier risk stratification (low/moderate/high risk) for fatal and non-fatal CVD. For primary efficacy evaluations, survival analysis was performed starting from enrolment to non-fatal and/or fatal CVD events up to the 5-year follow-up, or most recent follow-up. The cumulative incidence of CVD events of non-fatal and fatal disease was evaluated across 3 risk groups (low, moderate, and high) defined by Reti-CVD using the Kaplan–Meier method. Cox proportional hazards models were then used to estimate the adjusted HR trends (primary efficacy endpoint) by fitting a linear model to the 3-risk groups. We hypothesized that there is a trend in HR across the 3 ordinal risk groups and estimate the HR trend by considering the ordinal risk groups as continuous values of 1, 2, and 3. The *P*-value of the HR trend being less than 0.05 supports our hypothesis that risk stratification is possible. Adjusted HR trends were calculated after adjustment of age, gender, hypertension, hyperlipidemia, diabetes, and smoking status. Additionally, the performance in prediction of CVD events was assessed using Harrell’s C-index.

### Secondary efficacy endpoints

Secondary endpoints were evaluated using the HRs and HR trends for the following factors: (1) cardiac CT-measured CAC score-derived 3-tier cardiovascular risk (0, >0 to 100, and >100 Agatston unit); (2) CIMT from carotid ultrasonography-derived 2-tier cardiovascular risk (<90th percentile vs ≥90th percentile); and (3) baPWV-derived 2-tier cardiovascular risk (<1800 vs ≥1800 cm/s). Secondary efficacy evaluations were performed by entering the above covariates as independent factors into Cox proportional hazard models.

In addition, to test Reti-CVD as an independent risk predictor compared to other biomarkers, we used multivariable Cox models including Reti-CVD (3 groups), CAC score (3 groups), CIMT (2 groups), baPWV (2 groups), and other risk factors including age, gender, hypertension, diabetes, hyperlipidemia, and current smoker.

## Results

### Study population characteristics

Table 1 details the clinical characteristics of the participants according to the 3 risk groups stratified by the Reti-CVD risk groups (categorized as low-, moderate-, and high-risk groups). In the CMERC-HI, 33 (3.0%) had non-fatal and fatal CVD events during the 5-year follow-up. Non-fatal and fatal CVD events rate were 1.4% (9/646) in low-risk group, 3.8% (7/183) in moderate-risk group, and 6.1% (17/277) in high-risk group of Reti-CVD. Across these low-, moderate-, and high-risk Reti-CVD groups, the mean CAC scores were 130.2 (SD, 409.6), 310.3 (SD, 847.8), and 344.4 (SD, 628.4) Agatston unit, respectively.

**Table 1.** Characteristics of study population of CMERC-HI according to Reti-CVD risk groups (*n* = 1106).

	Reti-CVD risk groups		
Variables	Low	Moderate	High
Participants, *n* (%)	646	183	277
Cardiovascular events, *n* (%)	9 (1.4%)	7 (3.8%)	17 (6.1%)
Coronary artery calcium (CAC) score, mean (unit) (SD)	130.2 (409.6)	310.3 (847.8)	344.4 (628.4)
CAC-three risk groups			
Zero, *n* (%)	348 (53.9%)	54 (29.5%)	63 (22.7%)
>0 to 100, *n* (%)	156 (24.1%)	55 (30.1%)	78 (28.2%)
>100, *n* (%)	142 (22.0%)	74 (40.4%)	136 (49.1%)
Carotid intima media thickness, mean (mm) (SD)	0.7 (0.2)	0.8 (0.2)	0.8 (0.2)
<90th percentile, *n* (%)	589 (91.2%)	149 (81.4%)	212 (76.5%)
≥90th percentile, *n* (%)	57 (8.8%)	34 (18.6%)	65 (23.5%)
Brachial-ankle pulse wave velocity, mean (cm/s) (SD)	1430.3 (256.4)	1562.1 (266.1)	1636.1 (293.6)
<1800 cm/s, *n* (%)	592 (91.6%)	151 (82.5%)	210 (75.8%)
≥1800 cm/s, *n* (%)	54 (8.4%)	32 (17.5%)	67 (24.2%)
Age, mean (years) (SD)	54.8 (11.5)	63.7 (9.6)	67.0 (8.1)
Gender			
Male, *n* (%)	303 (46.9%)	115 (62.8%)	178 (64.3%)
Female, *n* (%)	343 (53.1%)	68 (37.2%)	99 (35.7%)
Systolic blood pressure, mean (mmHg) (SD)	125.8 (15.3)	130.3 (17.7)	130.6 (17.1)
Diastolic blood pressure, mean (mmHg) (SD)	77.6 (9.9)	75.4 (10.6)	74.0 (9.6)
Glucose, mean (mg/dL) (SD)	106.5 (26.3)	112.7 (25.7)	120.1 (37.8)
Hypertension, *n* (%)	596 (92.3%)	169 (92.3%)	259 (93.5%)
Diabetes, *n* (%)	196 (30.3%)	79 (43.2%)	144 (52.0%)
Dyslipidemia, *n* (%)	383 (59.3%)	113 (61.7%)	159 (57.4%)
Current smoker, *n* (%)	78 (12.1%)	25 (13.7%)	28 (10.1%)

Data are *n*, *n* (%), or mean (SD). CMERC-HI, Cardiovascular and Metabolic Disease Etiology Research Center-High Risk Cohort; CAC, coronary artery calcium.

### Performance of Reti-CVD in prediction of CVD event

**Figure 2.**
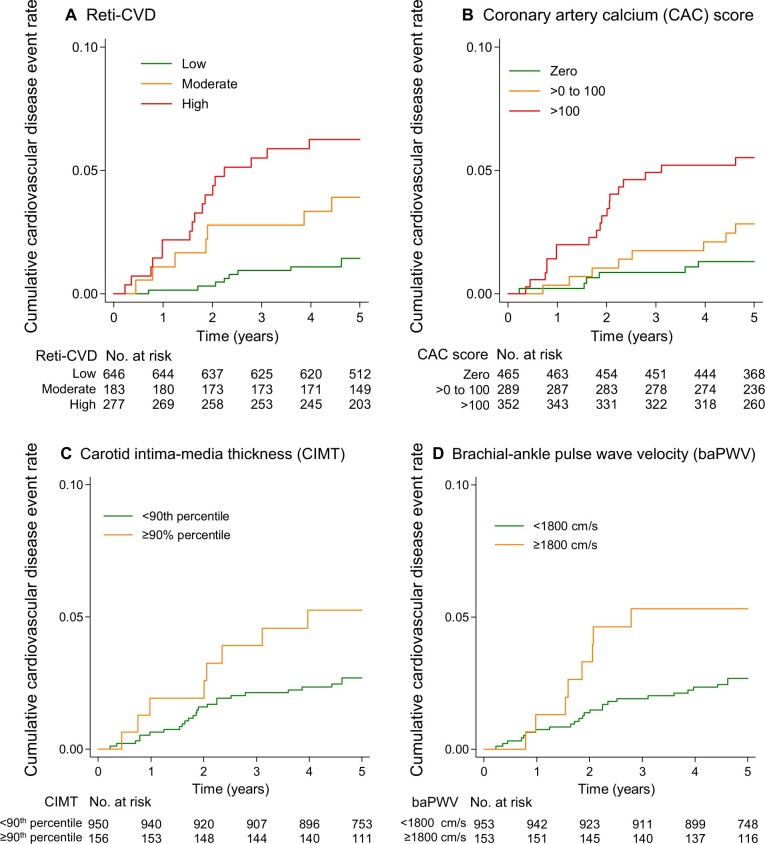
Kaplan–Meier estimates for cumulative incidence of cardiovascular events in the CMERC-HI cohort. Cardiovascular disease event according to Reti-CVD-three risk groups (A), coronary artery calcium (CAC)-based 3 risk groups (B), carotid intima-media thickness (CIMT)-based 2 risk groups (C), and brachial-ankle pulse wave velocity (baPWV)-based 2 risk groups (D). Cardiovascular disease events included incident heart failure, stroke, myocardial infarction, and cardiovascular mortality. CMERC-HI, Cardiovascular and Metabolic Disease Etiology Research Center-High Risk; CAC, coronary artery calcium; Reti-CVD, deep-learning-based retinal CVD score system; CIMT, carotid intima-media thickness; baPWV, brachial-ankle pulse wave velocity.

Kaplan–Meier curves were drawn according to the 3 Reti-CVD risk groups (Figure 2). During the 5-year follow-up, 5272.6 person-years were examined. Reti-CVD also shows distinct CVD risk stratification based on 3 groups (Figure 2A). CAC-three groups, and CIMT- and baPWV-two risk groups also show distinct CVD risk stratifications as shown in Figure 2.

Table 2 shows CVD risks according to Reti-CVD, CAC, CIMT, and baPWV using Cox regressions. The incidence of non-fatal and fatal CVD was 2.9 (95% CI, 1.5-5.5) in the low-risk group, 8.1 (3.8-16.9) in the moderate-risk group, and 13.3 (8.3-21.4) in the high-risk group according to Reti-CVD. The risk-adjusted HR trend (primary efficacy endpoint) was 2.02 (1.26-3.24) across 3 risk groups of Reti-CVD, indicating that the risk increases are doubled when a participant changes from one risk group to a one-step higher risk group. Here, the statistical significance of the *P*-value of the HR trend (p-trend) confirms our hypothesis that the Reti-CVD can stratify CVD risk.

**Table 2.** Risk of cardiovascular events by the Reti-CVD, and other biomarkers.

Outcomes/biomarker	*N*	Cases	Person-years	Incidence (95% CI)	Adjusted HR (95% CI)^b^	*P*-value
**Reti-CVD**						
Low	646	9	3129	2.9 (1.5-5.5)	1 (reference)	
Moderate	183	7	868	8.1 (3.8-16.9)	2.78 (0.95-8.11)	.061
High	277	17	1276	13.3 (8.3-21.4)	4.32 (1.62-11.54)	.003
HR trend^a^					2.02 (1.26-3.24)	.002*
C-statistics					0.751 (0.683-0.820)	
**CAC score**						
Zero	465	6	2243	2.7 (1.2-6.0)	1 (reference)	
>0 to 100	289	8	1394	5.7 (2.9-11.5)	1.86 (0.62-5.58)	.268
>100	352	19	1636	11.6 (7.4-18.2)	3.12 (1.13-8.60)	.027
HR trend					1.75 (1.08-2.84)	.025*
C-statistics					0.741 (0.673-0.807)	
**CIMT**						
<90th percentile	950	25	4547	5.5 (3.7-8.1)	1 (reference)	
≥90% percentile	156	8	725	11.0 (5.5-22.1)	1.52 (0.66-3.53)	.329
C-statistics					0.707 (0.623-0.791)	
**baPWV**						
<1800 cm/s	953	25	4561	5.5 (3.7-8.1)	1 (reference)	
≥1800 cm/s	153	8	712	11.2 (5.6-22.5)	1.43 (0.61-3.37)	.412
C-statistics					0.710 (0.625-0.794)	

a Primary efficacy endpoint.

b Risk-adjusted controlling for age group, gender, hypertension, dyslipidemia, diabetes, and smoking.

* *P*-value for trend.

Incidence per 1000 person-years. Cardiovascular event rates of cardiovascular disease events including incident heart failure, stroke, myocardial infarction, and cardiovascular mortality.

Delta from C-statistics between Reti-CVD and CAC score-based hazard model was 0.012 (−0.053 to 0.077, *P* = .723) for 5-year CVD events.

*N*, number at risk; HR, hazard ratio; CMERC-HI, Cardiovascular and Metabolic Disease Etiology Research Center-High Risk Cohort; CAC, coronary artery calcium; Reti-CVD, deep-learning retinal CVD score; CIMT, carotid intima-media thickness; baPWV, brachial-ankle pulse wave velocity.

Similar to Reti-CVD, the HRs of CVD events showed a dose–response association across the 3 risk strata according to the CAC score (risk-adjusted HR trend 1.75, 95% CI, 1.08-2.84). Based on CIMT- and baPWV-two risk groups, the risk-adjusted HRs for CVD events showed an association with HR of 1.52 (0.66-3.53), and 1.43 (0.61-3.37), which did not reach statistical significance.

The incremental value of the survival models in predicting cardiovascular risk was evaluated using Harrell’s C-index (Table 2). Harrell’s C-index were 0.751 (95% CI, 0.683-0.820) in the Reti-CVD model, 0.741 (0.673-0.807) in the CAC model, 0.707 (0.623-0.791) in the CIMT model, and 0.710 (0.625-0.794) in the baPWV model. In general, a C-index of 0.7 indicates a good model and 0.5 indicates that the model is no better at predicting outcome than random chance.

Lastly, when we incorporate all the biomarkers in one multivariable Cox model (Table 3), including Reti-CVD, CAC, CIMT, baPWV, and other traditional risk factors, the HRs by Reti-CVD were 2.40 (95% CI, 0.82-7.03) for the moderate-risk group, 3.56 (1.34-9.51) for the high-risk group compared to the low-risk group. CAC also showed a trend toward association with HR of 2.45 (0.88-6.84) in those with CAC > 100 compared to those with zero CAC; however, it was not statistically significant.

**Table 3.** Multivariable Cox model including Reti-CVD, other biomarkers, and traditional risk factors.

Outcomes/biomarker	Adjusted hazard ratio (95% CI)	*P*-value
**Reti-CVD score**		
Low	1 (reference)	
Moderate	2.40 (0.82-7.03)	.110
High	3.56 (1.34-9.51)	.011
**CAC score**		
Zero	1 (reference)	
>0 to 100	1.61 (0.53-4.87)	.395
>100	2.45 (0.88-6.84)	.086
**Carotid intima media thickness, mean (mm) (SD)**		
<90th percentile, *n* (%)	1 (reference)	
≥90th percentile, *n* (%)	1.50 (0.64-3.51)	.345
**Brachial-ankle pulse wave velocity, mean (cm/s) (SD)**		
<1800 cm/s, *n* (%)	1 (reference)	
≥1800 cm/s, *n* (%)	1.27 (0.53-3.03)	.589
**Age group (years)**		
20-44	1 (reference)	
45-49	0.84 (0.14-5.07)	.846
50-54	1.13 (0.25-5.00)	.873
55-59	0.21 (0.02-2.16)	.192
60-64	0.53 (0.11-2.68)	.446
65-69	0.56 (0.12-2.74)	.476
70-80	0.71 (0.16-3.20)	.652
**Other parameters**		
Female	0.72 (0.32-1.60)	.423
Hypertension	0.40 (0.15-1.07)	.068
Diabetes	0.96 (0.46-2.00)	.909
Hyperlipidemia	0.79 (0.39-1.61)	.521
Current smoker	1.47 (0.56-3.90)	.435

Cardiovascular event rates of cardiovascular disease events including incident heart failure, stroke, myocardial infarction, and cardiovascular mortality. HR, hazard ratio; CAC, coronary artery calcium; Reti-CVD, deep-learning retinal CVD score; CIMT, carotid intima-media thickness; baPWV, brachial-ankle pulse wave velocity.

## Discussion

In this retrospective analysis using prospectively collected clinical data of >1000 patients with 5 years of follow-up, we showed that the AI algorithm, Reti-CVD, positioned as an AI-SaMD, predicted future non-fatal and fatal CVD events and stratified patients into 3 distinct risk groups (low-, moderate-, and high-risk) effectively. We evaluated whether CVD risk could be stratified into 3 distinct risk groups using Reti-CVD, with a pre-specified primary efficacy endpoint of CVD events and evaluated as the HR trend increase in CVD risk. HR trend was 2.02 (95% CI, 1.26-3.24) across the Reti-CVD risk groups. In a pre-specified secondary analysis, we compared with other CVD biomarkers for CVD risk stratification, demonstrating Reti-CVD showed non-inferior performance to CAC score-based risk stratification, and superior performance compared to CIMT and baPWV.

### Rationale for regulatory approval

Our study design is unique in that it utilized survival analysis and the primary efficacy endpoint was evaluated by HR trend. Area under the receiver operating characteristic, sensitivity, or specificity is typically used in other AI-SaMD to determine the presence or absence of disease in cross-sectional evaluation. As Reti-CVD is a biomarker that stratifies future risk rather than diagnosing current disease, its performance should be evaluated in survival analysis through longitudinal studies.

Our study design is also unique in that we performed retrospective verification. In the guidelines provided by the K-MFDS in July 2022,^17^ retrospective data can be used if there is data that meet the purpose of use when evaluating AI-SaMD. The US Food and Drug Administration (FDA) also allows “existing data” to be used for verification, in the following 2 cases: (1) Referencing existing data from studies conducted for the same intended use; (2) Referencing existing data from studies conducted for a different intended use, where extrapolation of such data can be justified.^18^ Otherwise, current guidelines recommend that studies generate new clinical data for a specific intended use.^18^ However, in the case of AI-SaMDs identifying diabetic retinopathy with retinal photographs that have been approved so far (ie, LumineticsCore [former IDx-DR], Digital Diagnostics; EyeArt, Eyenuk; and AEYE-DS, AEYE Health), pivotal studies have been newly performed.

With regard to using retrospective validation and HR as primary endpoints, there exists a precedent of FDA approval. Specifically, “DiaDexus” employed plasma samples from the Atherosclerosis Risk in Communities (ARIC) study, an initiative begun in 1985, which delved into the causes and clinical implications of atherosclerosis. Through this data, “DiaDexus” evidenced the efficacy of the PLAC test, a diagnostic tool measuring Lipoprotein-Associated Phospholipase A2 (Lp-PLA2) in plasma, which aids in gauging the risk of coronary heart disease and ischemic stroke. This methodology secured FDA endorsement, and, to our knowledge, stands as an emblematic instance of product licensing based on retrospective data with HR as the primary determinant. A detailed comparison between Lp-PLA2 and Reti-CVD can be found in Appendix S7.

The approval was possible with a single-center design because of our robust generalizable clinical validation through peer-reviewed publications as independent reviews. According to the International Medical Device Regulators Forum (IMDRF), independent review of clinical evidence is critical for regulatory approval of AI-SaMDs.^19^ Independent review includes formal consultation with regulators, third parties on behalf of regulators, or the editorial board of a peer-reviewed journal. We provided robust clinical evidence via peer-reviewed articles.^13^^,^^15^ These publications showed that Reti-CVD was validated previously based on the UK Biobank (*n* = 47 679), and the Singapore Epidemiology of Eye Diseases study (*n* = 8551) including Malay, Indian, and Chinese ethnicities.^13^

Reti-CVD is considered as low/moderate impact SaMD category according to the IMDRF.^19^ Our intended target population is asymptomatic adults who have not been previously diagnosed with CVD, suggesting that individuals may not always be patients, and clinical information provided by Reti-CVD will not trigger an immediate action for acute CVD. Reti-CVD provides the probability of future CVD events rather than diagnosing CVD such as acute stroke or myocardial infarction, indicating Reti-CVD’s purpose to drive clinical management on “non-serious conditions” rather than “serious conditions.” Moreover, Reti-CVD may not only encourage early intervention for current CVD “risk factors,” such as unhealthy lifestyles, obesity, and known chronic conditions; but also promote evaluation for unknown chronic conditions such as hypertension, hyperlipidemia, and diabetes in conjunction with clinical evaluation.

Thus, on the basis of the aforementioned rationales, the K-MFDS approved Reti-CVD based on retrospective survival analysis using prospectively collected data, which was collected in single center. The strength of this study is that we reported the first regulated pivotal study on AI-SaMD with an intended use for future disease risk stratification rather than assisting diagnoses.

### Practical real-world workflow and reimbursement

In earlier study, the application of Reti-CVD to the existing 10-year ASCVD risk estimators (European SCORE and US Pooled Cohort Equation) resulted in an increase in the net reclassification index by 22%-26%.^13^ Consequently, the combined approach enhances the effectiveness of identifying patients at risk. Use of point-of-care CVD risk triage with the Reti-CVD is especially helpful for identifying at-risk groups in CVD primary prevention because the retinal camera is easy to operate, highly portable, and has a smaller footprint. Therefore, using an in-house retinal camera in primary care, with on-site personalized CVD risk assessment that is comparable to CT-measured CAC, improves clinical workflow as it: (1) lessens the need for secondary imaging referral, (2) enables same session patient–doctor shared decision-making, (3) provides additional risk-enhancing factor for patients who are hesitant on starting pharmacotherapy, and (4) avoids delays in clinical intervention for actual at-risk patients. Moreover, compared to the clinical recommendation for cardiac CT scanning intervals of 3-5 years, Reti-CVD allows for more frequent patient re-screenings given that it is non-invasive, harmless with no radiation risk, and fast with a simplified workflow.^7^^,^^20^

In terms of reimbursement, doctors can diagnose eye diseases from retinal photos, but predicting cardiovascular risk is a unique area that only AI can do (ie, humans cannot quantify CVD risk based on retinal photos). Therefore, Reti-CVD SaMD was classified as a medical device that needs to be evaluated as a “new medical technology” despite the use of an already-approved medical device, the retinal camera. This was decided by the Korean Health Insurance Review & Assessment (HIRA, similar to US Centers for Medicare & Medicaid Services [CMS]) and will be evaluated by National Evidence-based healthcare Collaborating Agency (NECA, similar to US American Medical Association [AMA]).

Recently, a new policy is being implemented to launch AI and digital medical device quickly into the market through an “innovative medical technology track” or “exemption (deferral) track for a safe device (eg, non-invasive tool)” in Korea. This is similar to the breakthrough medical device designation by the US FDA. As retinal photography can be obtained non-invasively, Reti-CVD is under evaluation for the aforementioned exemption track. Once NECA (∼ AMA) grants an exemption track, a new medical device will receive a temporary code for 2 years and be reimbursed by private insurers, which covers ∼70% of the Korean population. However, the National Health Insurance Service (NHIS), a government sponsored single payer insurance system, will not reimburse costs related to this device. Currently, Reti-CVD is covered by private insurance providers in South Korea. Its use is extending from ophthalmology to frontline care in both familiy and internal medicine.

## Conclusions

Thus, after a comprehensive review of the clinical evidence and benefit/risk analysis, K-MFDS approved the use of Reti-CVD via a single centered trial. These results led K-MFDS to authorize Reti-CVD as a retinal image-based, personalized CVD risk scoring system, which is designed to aid healthcare professionals identify and manage current CVD risk factors, in addition to clinical evaluation, among asymptomatic adults who have not been previously diagnosed with CVD. From a broader perspective, these results demonstrate the ability of deep learning to bring automated CVD risk quantification which cannot be performed by humans.

## Supplementary Material

ocad199_Supplementary_Data

## Data Availability

Data cannot be shared publicly due to the violation of patient privacy and the absence of informed consent for data sharing. Data from CMERC-HI are available to researchers who meet the criteria for access to confidential data; requests should be made to Prof. Sungha Park (SHPARK0530@yuhs.ac).
