# Supplementary material for: Pivotal trial of a deep-learning-based retinal biomarker (Reti-CVD) in the prediction of cardiovascular disease: data from CMERC-HI
Source: J Am Med Inform Assoc. 2023 Oct 17;31(1):130–8. doi: 10.1093/jamia/ocad199 (PMC10746299; doi:10.1093/jamia/ocad199)
Supplement: ocad199_Supplementary_Data [file ocad199_supplementary_data.pdf]

Appendix

Appendix 1. Use of CMERC-HI data in previous and current studies

The Cardiovascular and Metabolic Disease Etiology Research Center–High Risk (CMERC-HI) Study is a prospective observational cohort study comprising patients at a high risk of developing CVD who do not have symptomatic CVD at baseline (Clinicaltrials.gov identifier: NCT02003781).

Our article unveiled our initial Poof-of-concept (PoC) with retina and deep learning for cardiovascular risk stratification (Reti-CVD). This revealed its potential to forecast future cardiovascular diseases. The paper expounded on our algorithm development process and its verification using three distinct longitudinal datasets: interim data of CMERC-HI (2013-2016), Singapore data, and UK Biobank data.

This current study narrates the clinical trial process and the results pursued for AI-SaMD regulatory approval, following the commercialization of the deep learning algorithm curated in our PoC study. While the data mirrors the CMERC-HI from our initial PoC research, it envelops a comprehensive dataset collected between 2013-2018, effectively doubling the subject count compared to prior studies utilizing interim data (2013-2016, **Table** below. Notably, our subject count was finalized post sample size calculation during the clinical trial's design phase. Whereas the initial PoC study juxtaposed only retinal photo with cardiac CT CAC, this research compares performance inclusive of cardiac CT CAC, CIMT, and BaPWV—secondary endpoints in the clinical trial. Of paramount importance is the distinction that, while existing AI SaMDs cater to diagnosis, this AI-SaMD stands as a prognostic biomarker, forecasting impending risks. The unveiling of this pioneering AI-SaMD's regulatory clearance journey is of profound global significance.

Table. Use of CMERC-HI in previous proof-of-concept studies and in current clinical trials

Variable	Previous PoC	Current clinical trial
Purpose of study	Proof of concept (PoC)	Regulatory approval
Study design pre-confirmation	IRB	The Ministry of Food and Drug Safety, and IRB
Clinical trial registration	Registered as original study (NCT02003781)	In addition, separate registration done as a clinical trial for regulatory approval (KCT0007047)
Study period setting	Interim data (2013-2016)<br>(The last follow-up date: Dec 31, 2018)	Comprehensive data (2013-2018)<br>(The last follow-up date: Dec 31, 2020)
Modalities	Reti-CVD vs coronary artery calcium (CAC)	Reti-CVD vs [CAC, CIMT, BaPWV]
Sample size	527	1106 based on sample size calculation

Appendix 2. Definition of cardio cerebral vascular disease

Heart failure (HF) hospitalization was defined as an event that meets all of the following criteria: ***1)*** requiring hospitalization due to clinical manifestations of HF (e.g., dyspnoea, orthopnoea, paroxysmal nocturnal dyspnoea, oedema, pulmonary basilar crackles, jugular venous distension, third heart sound or gallop rhythm, radiological evidence of worsening HF) and additional therapy including an oral or intravenous diuretic, inotrope, or vasodilator therapy, and ***2)*** elevated serum biomarker (N terminal pro-brain natriuretic peptide) level >300 ng/L (if there was evidence that left ventricular ejection fraction was <40% in any imaging modality including echocardiography, myocardial perfusion scan, or cardiac magnetic resonance imaging, a threshold of 600 ng/L was used).[22]

Non-fatal myocardial infarction was used when there was evidence of myocardial necrosis in a clinical setting consistent with myocardial ischemia. It was defined as a patient admitted for myocardial infarction with at least two of the following three criteria: ***1)*** symptom of ischemic chest pain, ***2)*** elevation of cardiac enzyme level and ***3)*** significant luminal narrowing of coronary artery confirmed by any imaging modality including angiography or computed tomography scan.

Stroke was defined as a composite of haemorrhagic stroke and ischemic stroke requiring hospitalization due to new-onset neurologic deficit and correlating lesions found in brain imaging studies.

Appendix 3. Reti-CVD score development and rationale

Developmental process and hypothesis

Details of deep learning algorithm regarding model development has been described elsewhere.[13] Overall development process of Reti-CVD and training/validation sets are described in the figure below. First, we used the Korean health screening data for training. The model input was retinal photographs, and we trained our deep-learning algorithm to predict coronary artery calcium (CAC, binary class of presence versus absence). Here, in typical deep learning research, the approach is to test how well the deep learning algorithm predicts the ground truth of CAC with the area under the receiver operating characteristic (AUROC), sensitivity, or specificity. In this earlier study, we found that this "deep-learning-based probability score (=Reti-CVD score)" could predict future CVD events in longitudinal study rather than just predict CAC in a cross-sectional study. Thus, the "Reti-CVD score" was designed to calibrate the amount of association between retinal microvascular signs and the presence of CAC, which is a robust predictor of CVD risk. Finally, we confirmed that the Reti-CVD score could stratify future CVD risk in three external longitudinal studies (time-to-events data) including Korean, Singaporean Malay/Indian/Chinese, and British White cohorts.[13] In the current study, the potential for overfitting is negated as the validation leveraged the CMRC-HI dataset—a dataset distinctly separate from the health screening center data. We crafted the deep learning algorithm using cross-sectional screening data, and its validation as a novel biomarker was carried out through survival analysis with the CMERC-HI cardiovascular cohort, which was curated for a different objective. Hence, the data used in this validation is wholly distinct from that of the deep learning process, ensuring the absence of overfitting.

Deep learning training

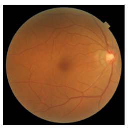

Coronary artery calcium (CAC)

Presence versus Absence

Input **Reference Standard (Ground Truth)**

Training data: Korean Health Screening data
(Rim et al., The lancet Digital Health, 2021)

Hypothesis

This "deep-learning-based probability score (=Reti-CVD score)" can be used as a new biomarker that can stratify the risk of incident CVD among people without CVD at baseline.

Rather than predicting the presence of CAC cross-sectionally [typical deep learning approach]

Validation

Survival analysis using the longitudinal study (time-to-event data) to confirm Reti-CVD score can be used as risk stratification tool

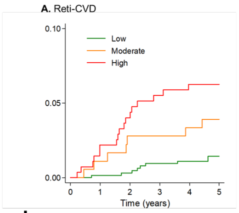

Longitudinal data: CMERC-HI

CMERC-HI: Cardiovascular and Metabolic Diseases Etiology Research Center-High Risk Cohort

Direct training versus indirect training

While direct training on CVD events is possible, direct prediction of events presents some difficulties: 1) training can be difficult due to the small number of CVD events, 2) it is difficult to properly consider the time effect in the training process, and finally 3) it is greatly influenced by the characteristics of the developed set (race, region, health information system, hospital, ICD code, etc.). Thus, developing general models can be difficult.

Compared to direct training on CVD events, there are advantages of indirect training: 1) we could use a large dataset, which included retinal photograph-CAC paired data from health screening data, 2) Reti-CVD could be developed cross-sectionally and time effect could therefore be clearly separated and considered in survival analysis, and 3) CAC is mainly calculated by automated computer programs, so no human errors (such as miss diagnosis in claim data) are involved in labelling for ground truth.

For example, in another earlier study from our group, we developed a retina-based biological age (termed Reti-Age) based on a deep learning algorithm trained using retinal photos.[1] We trained the DL algorithm to predict the probability for an individual of being ≥65 years old (ground truth: <65 versus ≥65 years old) based on retinal photos using data from a health-screening centre in Korea. Reti-Age was associated with all-cause, cardiovascular disease and cancer mortality, and with cardiovascular and cancer events, independently of chronological age and phenotypic biomarkers. Then we concluded that this approach provides a novel, alternative approach to measure biological age using retinal photographs.

Deep learning model updates

Details of deep learning algorithm development and model updates has been described elsewhere. In a previous study, there were two large health examination datasets from Severance Hospital in Korea (Dataset 1) and Phillip Medical Center in Korea (Dataset 2) and included retinal images and cardiac CT scans taken on the same day. In the initial development, the algorithm was developed using set 1, and for model updates, both sets 1 and 2 were used to maximize the amount of training set.

Also, typical classification ConvNets consist of a feature extractor and a classification layer.

---

[1] Retinal photograph-based deep learning predicts biological age, and stratifies morbidity and mortality risk; S Nusinovici, TH Rim, M Yu, G Lee, YC Tham, N Cheung, CCY Chong, ...; Age and ageing 51 (4), afac065

The model extracts discriminant features through convolutional neural network (CNN) filters, computes probabilities, minimizes target and prediction loss, and updates parameters through back-propagation in an end-to-end fashion. This process allows our model to find optimal parameters in a non-manual way. Our deep learning model is based on a convnext model that outperforms previous CNN models.

This updated Reti-CVD showed higher AUC of 0.779 (95% CI, 0.768 - 0.790) in the internal test set than that of the previous AUC of 0.731 (95% CI, 0.712-751).

Three-tier risk stratification system

The Reti-CVD score was defined based on a probability score derived from our deep-learning algorithm of binary classification (absence **vs** presence of CAC). The probability scores ranged from zero to one (as a continuous variable), with a high value indicating a high probability of the presence of CAC. The distribution of the estimated Reti-CVD scores is provided below. We proposed a new cardiovascular disease risk stratification system based on the tertiles of Reti-CVD scores in CMERC-HI interim data (1st tertile ≥0 to <0.3093, low risk; 2nd tertile ≥0.3093 to ≤0.4074, moderate risk; and 3rd tertile >0.4074 to ≤1, high risk). We used these proposed cutoff values to further stratify the CVD event in this pivotal study.

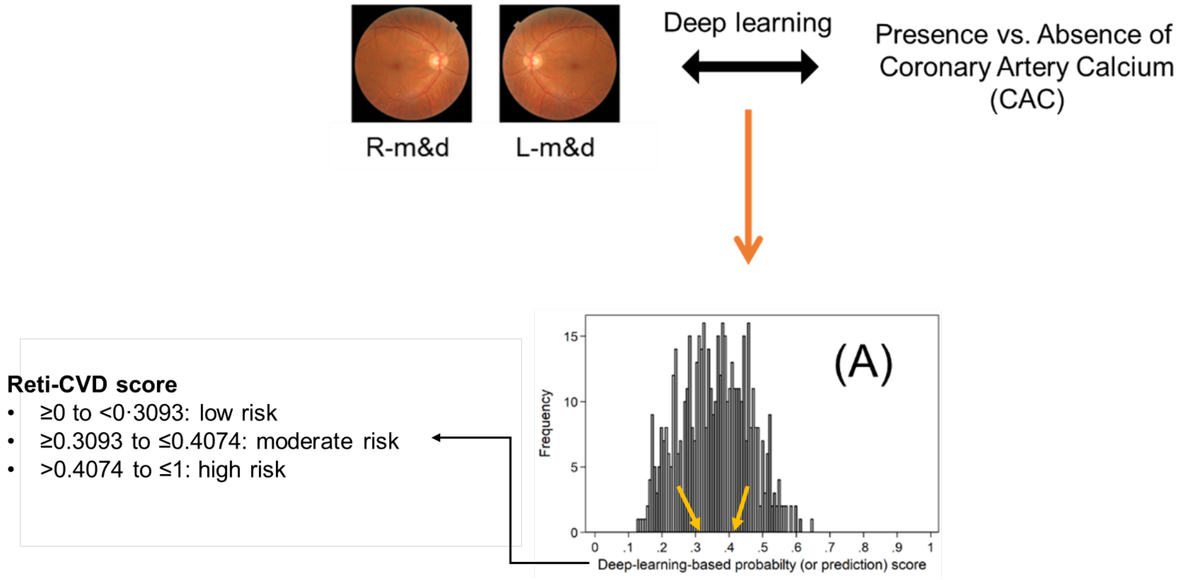

Appendix 4. Measurement of cardiovascular biomarkers

Carotid intima-media thickness (CIMT): Both common carotid arteries were scanned by a single expert operator with a high-resolution real-time 8-MHz linear scanner (Acuson300, Siemens, Munich, Germany). An optimal longitudinal B mode image of both common carotid arteries was obtained in the lateral plane and stored digitally. The IMT of far wall was measured along 10-mm proximal to bifurcation using an automated edge detection algorithm and dedicated software (Syngo AHP, Siemens, Munich. Germany). The largest IMT value was recorded as the maximum IMT (max-IMT). The mean IMT (mean-IMT) was determined as the average value of measured IMT. The average of right and left values was used as the individual representative value. Carotid plaque was defined as echo-structure encroaching into the lumen by more than 50% thickening of the surrounding area or with absolute thickness of >1.5 mm.

Brachial-ankle pulse wave velocity (baPWV): baPWV was measured using a volume-plethysmography device (OMRON, Tokyo, Japan). The patients were examined while resting in the supine position. Electrocardiographic electrodes were placed on both wrists, and cuffs were wrapped on both arms and ankles. Pulse volume waveforms at both brachial and posterior tibial arteries were recorded using a semiconductor pressure sensor after patients rested for at least 5 min. The heart rate was continuously recorded with flow and pressure tracings gated to the electrocardiogram, and baPWV was calculated automatically using time-phase analysis. The distance between the upper arm and ankle was estimated based on height. We used the average baPWV from right and left measurements in the analysis. Brachial pulse pressure was calculated using the systolic and diastolic blood pressures derived during baPWV measurement.

Coronary artery calcium (CAC) score: The patients were scanned using a 320-detector row CT scanner (Aquillion ONE; Toshiba Medical Systems Corporation, Tokyo, Japan). A non-enhanced prospective electrocardiogram-gated CT scan was performed to measure the CAC score with the following parameters: rotation time 275 ms, slice collimation 0.5 mm, slice thickness 3.0 mm, tube voltage 100 kV, and automatic tube current modulation (SURE Exposure 3D standard; Toshiba Medical Systems Corporation, Otawara, Japan). Images were analysed on a core workstation by using dedicated software (TeraRecon V. 4.4.11.82.3430. Beta, Foster City, CA, USA). CAC score was calculated using the reduced tube voltage protocol,[2] and the CAC score was further checked by a radiology-trained cardiologist.

---

[2]Nakazato R, Dey D, Gutstein A, Le Meunier L, Cheng VY, Pimentel R, Paz W, Hayes SW, Thomson LE, Friedman JD. Coronary artery calcium scoring using a reduced tube voltage and radiation dose protocol with dual-source computed tomography. Journal of Cardiovascular Computed Tomography 2009;3:394-400.

Appendix 5. Sample size calculation

Planned Sample Size

	Investigational Medical Device*	Total Subjects
No. Subjects	1050	1050
No. Subjects (including 5% drop-out rate)	1106	1106

Planned Sample Size and Size Determination

Hypothesis 1: The retinal fundus photography-based risk assessment software tool, "Reti-CVD", will be able to differentiate individuals into a 3-tier risk stratification (low/moderate/high risk) for fatal and nonfatal cardiovascular disease, and this 3-tier risk classification will show good concordance with clinical outcomes in a large Korean cohort with 5 years' follow-up data.

$$H_0 : \theta = 1 \qquad \text{v.s.} \qquad H_1 : \theta \neq 1$$

Primary Efficacy Endpoint: Cox proportional hazards models were then used to estimate the hazard ratios (HRs) and HR trends by fitting a linear model to the three risk categories. The HR trends were used to evaluate the primary efficacy endpoint.

Statistical Assumptions

1) Level of significance, α = 0.05, Zα/2 = 1.96
2) Type 2 error(β) = 0.2; power of the test = 80%.
   1-β = 0.8, = 0.842
3) $\theta = 2.02$ $\quad \psi = 0.032$ $\quad \sigma^2 = 0.67$ $\quad \rho^2 = 0.34$
   - The following outcomes data were used as reference: In a preceding study (Rim et al, The Lancet Digital Health, 2021), a total of 527 subjects were followed-up for 5 years with a cardiovascular event rate of 3.2%; the estimated high-/low-risk groups stratified by Reti-CVD showed that, relative to the low-risk group, the high-risk group had a hazard ratio (HR) of 4.10 (95% CI, 1.22-13.77) and an HR trend of 2.02 (95% CI, 1.18-3.46).
   - θ=2.02: calculated HR trend
   - ψ=0.032: 3.2% event rate
   - σ^2 =0.67: 3-strata risk variance
   - ρ^2 =0.34: calculated R2 value using regression models with the retinal image-based scores as a biomarker in addition to traditional risk factors including age, sex, smoking status, hypertension, diabetes, and hyperlipidemia

Determination of the Planned Sample Size: The sample size of 1050 subjects was calculated using the R package “powerSurvEpi”, with a 5% significance level, an assumed HR trend of 2.02 and 80% power for non-one HR detection. (Hsieh & Lavori, 2000)*

$$n = \frac{\left(z_{\alpha/2} + z_{\beta}\right)^2}{[\log(\theta)]^2 \sigma^2 \psi (1 - \rho^2)}$$

Where θ represents the hazard ratio trend for the covariate of interest, σ^2 represent the variance of covariate of interest, ρ^2 represents the R-squared of the regression of interested covariate on other covariates adjusted in the Cox proportional-hazards model, ψ represents the proportion of subject died, z_(α/2) and z_β represent the critical values of standard normal distribution corresponding to the required significance level of α and power of (1-β).

Among the calculated sample size of 1050 subjects, a drop-out rate of 5% was assumed, resulting in the total planned subject number of 1106. Thus, after retrospectively reviewing the cohort data for inclusion/exclusion, a total of 1106 subjects were planned to be enrolled for the analysis. If a greater number of subjects were eligible for enrollment after inclusion/exclusion than the planned sample size (n=1106), then randomization for further exclusion would be performed to select the target number of 1106 subjects for analysis. Please see **Appendix 5**.

Appendix 6. Randomization method for further exclusion

If a greater number of subjects were eligible for enrollment after inclusion/exclusion than the planned sample size (n=1106), then randomization for further exclusion would be performed to select the target number of 1106 subjects for analysis according to the following steps in those eligible subjects:

1) Subjects were numbered consecutively from the date of enrollment to the original cohort.
2) An independent statistician (with no conflicts of interest) performed randomization using SAS Version 9.4 (or latest).
3) The randomized numbers and subject numbers were matched 1:1.
4) The randomized numbers were ordered in descending order (lowest number first), and then numbers 1 through to 1106 were selected for inclusion in the current study.
5) The randomization and selection process was documented and kept for recording.

Example) 3 selected among 5 eligible subjects

Enrolment Date / No.		Randomized Number (by SAS)
2013-11-01	01	03
2013-11-02	02	04
2013-11-04	03	02
2013-11-06	04	05
2013-11-07	05	01

➔ Enrolment numbers 05, 03, 01 selected for inclusion

Appendix 7. Point-by-point comparison between Lipoprotein-associated phospholipase A2 and Reti-CVD

Biomarker	Lipoprotein-associated phospholipase A2 (Lp-PLA2)<br>(Product name: diaDexus™ PLAC™ Test)*	Reti-CVD score<br>(Product name: DrNoon for CVD in Korea; Reti-CVD in outside of Korea)
Indication for use	The diaDexus PLAC™ test is an enzyme immunoassay for the quantitative determination of Lp-PLA2 (lipoprotein-associated phopholipase A2) in human plasma, to be used in conjunction with clinical evaluation and patient risk assessment as an aid in predicting risk for coronary heart disease (CHD).	Artificial intelligence-based software that analyzes the structure of the retina and the shape of blood vessels in the retinal photographs of patients with suspected cardiovascular risk and displays the degree of cardiovascular disease risk (Low: low risk, Moderate: moderate risk, High: high risk) with score (=Reti-CVD score).
Pivotal dataset	Atherosclerosis Risk in Communities (ARIC)<br>A government funded prospective study to investigate the etiology of atherosclerosis and its clinical sequelae and variation in cardiovascular risk factors, medical care, and disease by race, sex, place, and time.<br>1348 participants between 47 and 69 years old and a 9-year follow-up study.	Cardiovascular and Metabolic Diseases Etiology Research Center-High Risk Cohort (CMERC-HI)<br>A government funded prospective study to investigate specific preventive strategies for patients with a high risk of CVD.<br>1106 participants between 20 and 80 years old, and a 10-year follow-up study.
Definition of risk strata	Used high and low cutpoints of LpPLA2, generated from the study (420 and 310 ng/mL, the 67th and 33rd percentiles, respectively)	Used pre-specified thresholds, which was published in an earlier study.<br>RetiCAC scores-based 3 risk groups**<br>▪ ≥0 to <0.3093: low<br>▪ ≥0.3093 to ≤0.4074: moderate<br>▪ >0.4074 to ≤1: high

Primary efficacy endpoint	**Hazard ratio (HR)**<br><br>The hazard ratios of the Cox regression analyses demonstrated that Lp-PLA2 may be used as a predictor of risk for CHD, for the highest and intermediate levels when compared to the lowest level of Lp-PLA2, (see Table below***).	**Hazard ratio (HR) trend**<br><br>For primary efficacy evaluations, survival analysis was performed starting from enrolment to nonfatal and/or fatal cardiovascular disease events up to the 5-year follow-up, or most recent follow-up. The cumulative incidence of CVD events of non-fatal and fatal disease was evaluated across 3 risk groups (low, moderate, and high) defined by Reti-CVD using the Kaplan-Meier method. Cox proportional hazards models were then used to estimate the adjusted **HR trends (primary efficacy endpoint)** by fitting a linear model to the three-risk groups. Adjusted HR trends were calculated after adjustment of age, gender, hypertension, hyperlipidaemia, diabetes, and smoking status.”

*510(k) SUBSTANTIAL EQUIVALENCE DETERMINATION DECISION SUMMARY DEVICE ONLY TEMPLATE
** Deep-learning-based cardiovascular risk stratification using coronary artery calcium scores predicted from retinal photographs; TH Rim, CJ Lee, YC Tham, N Cheung, M Yu, G Lee, Y Kim, DSW Ting, ...; The Lancet Digital Health 3 (5), e306-e316
***Primary efficacy endpoint for Lp-PLA2

Table 1. Risk Ratios of CHD for Subjects Across All LDL Levels

	Lp-PLA2 Risk Ratio (95% CI, p-value)		
Lp-PLA2 (ng/mL)	<310	310-420	>420
Model 1	1.0*	1.49 (1.11-1.99, p=0.008)	2.50 (1.89-3.31, p<0.001)
Model 2	1.0	1.24 (0.92-1.66, p=0.154**)	1.76 (1.32=2.36, p<0.001)
Model 3	1.0	1.71 (1.06-2.75, p=0.029)	2.12 (1.29-3.48, p=0.003)

*The lowest tertile with Lp-PLA2 values <310 ng/mL is used as the reference group
**p-value is not significant

Model 1: univariate analysis
Model 2: adjusted for age, race, gender
Model 3: adjusted for age, race, gender, current smoking status, blood pressure, diabetes, HDL, LDL, CRP and Lp-PLA2 - LDL interaction
